# Supplementary material for: Does food insecurity cause anxiety and depression? Evidence from the changing cost of living study
Source: PLOS Ment Health. 2025 Jul 16;2(7):e0000320. doi: 10.1371/journal.pmen.0000320 (PMC12798610; doi:10.1371/journal.pmen.0000320)
Supplement: S1 Text — Departures from preregistration, supplementary tables, supplementary figure. (PDF) [file pmen.0000320.s001.pdf]

# Supplementary Information for ‘Does food insecurity cause anxiety and depression? Evidence from the Changing Cost of Living Study’

**Table A.** Departures from preregistration.

| | **Departure from preregistration** | **Rationale** |
|---|---|---|
| Models 1-3 | Month and lagged mental health scores were added as additional covariates. | The rationale for including month was that food insecurity, anxiety and depression all declined over the course of the study leading to month being a potential confound. The rationale for including lagged mental health was the high within-individual consistency in mental health measures in the dataset. |
| Model 4 | Omitted. | Not required, since there was no evidence for differential effects of food insecurity on anxiety and depression in Model 1. |
| Models 5 and 6 | Rather than focussing solely on within-individual effects in these models, we used the method proposed by Bolger & Laurenceau (2013) to decompose food-insecurity status into between- and within-individual components and included both components in our models as predictors. Since our models now contained between-individual effects we included gender, age, month and lagged mental health status as covariates replicating the approach we took in Models 1-3. | The Bolger & Laurenceau (2013) method is an improvement over the approach we preregistered because: (1) it allowed us to model all of the data, and (2) it allowed us to compare the size of between- and within-individual effects of food insecurity. We were unaware of this method when we wrote the preregistration. |

**Table B.** Output of LMMs predicting prevalence of food insecurity, GAD-7 and PHQ-8 scores by month of the study.

| | **FI status** | | | **GAD-7 score** | | | **PHQ-8 score** | | |
|---|---|---|---|---|---|---|---|---|---|
| *Predictors* | *Odds Ratios* | *CI* | *p* | *Estimates* | *CI* | *p* | *Estimates* | *CI* | *p* |
| (Intercept) | 0.00 | 0.00 – 0.01 | **<0.001** | 5.20 | 4.59 – 5.82 | **<0.001** | 6.09 | 5.44 – 6.73 | **<0.001** |
| Month | 0.83 | 0.71 – 0.98 | **0.031** | -0.05 | -0.08 – -0.02 | **<0.001** | -0.09 | -0.12 – -0.06 | **<0.001** |
| Gender: Woman | 2.03 | 0.76 – 5.37 | 0.155 | 1.23 | 0.38 – 2.08 | **0.005** | 0.83 | -0.06 – 1.71 | 0.068 |
| Gender: PNTS or self-describe | 0.25 | 0.00 – 156.44 | 0.675 | 1.48 | -2.88 – 5.85 | 0.505 | 3.22 | -1.35 – 7.78 | 0.167 |
| Participants | 483 | | | 483 | | | 483 | | |
| Observations | 4860 | | | 4844 | | | 4851 | | |

**Table C.** Output of LMMs to test whether food insecurity Granger causes GAD-7.

| | GAD-7 (univariate model) | | | GAD-7 (bivariate model) | | |
|---|---|---|---|---|---|---|
| *Predictors* | *Estimates* | *CI* | *p* | *Estimates* | *CI* | *p* |
| (Intercept) | -0.10 | -0.19 – -0.02 | **0.018** | -0.12 | -0.20 – -0.03 | **0.008** |
| age | -0.02 | -0.02 – -0.01 | **<0.001** | -0.02 | -0.02 – -0.01 | **<0.001** |
| gender [Woman] | 0.20 | 0.08 – 0.32 | **0.001** | 0.19 | 0.07 – 0.31 | **0.001** |
| gender [PNTS or self-describe] | 0.25 | -0.51 – 1.02 | 0.520 | 0.27 | -0.49 – 1.02 | 0.492 |
| month | -0.01 | -0.01 – -0.00 | **0.001** | -0.01 | -0.01 – -0.00 | **0.001** |
| lagged GAD | 0.24 | 0.21 – 0.27 | **<0.001** | 0.24 | 0.21 – 0.27 | **<0.001** |
| lagged FI status | | | | 0.08 | 0.03 – 0.14 | **0.005** |
| Participants | 481 | | | 481 | | |
| Observations | 4347 | | | 4347 | | |

**Table D.** Output of LMMs to test whether food insecurity Granger causes PHQ-8.

| | **PHQ-8 (univariate model)** | | | **PHQ-8 (bivariate model)** | | |
|---|---|---|---|---|---|---|
| *Predictors* | *Estimates* | *CI* | *p* | *Estimates* | *CI* | *p* |
| (Intercept) | -0.07 | -0.15 – 0.01 | 0.106 | -0.08 | -0.16 – 0.01 | 0.072 |
| age | -0.01 | -0.02 – -0.01 | **<0.001** | -0.01 | -0.02 – -0.01 | **<0.001** |
| gender [Woman] | 0.13 | 0.01 – 0.25 | **0.027** | 0.13 | 0.01 – 0.25 | **0.029** |
| gender [PNTS or self-describe] | 0.53 | -0.22 – 1.28 | 0.165 | 0.54 | -0.20 – 1.28 | 0.155 |
| month | -0.01 | -0.02 – -0.01 | **<0.001** | -0.01 | -0.02 – -0.01 | **<0.001** |
| lagged PHQ | 0.26 | 0.23 – 0.29 | **<0.001** | 0.26 | 0.23 – 0.29 | **<0.001** |
| lagged FI status | | | | 0.05 | -0.01 – 0.11 | 0.090 |
| Participants | 480 | | | 480 | | |
| Observations | 4363 | | | 4363 | | |

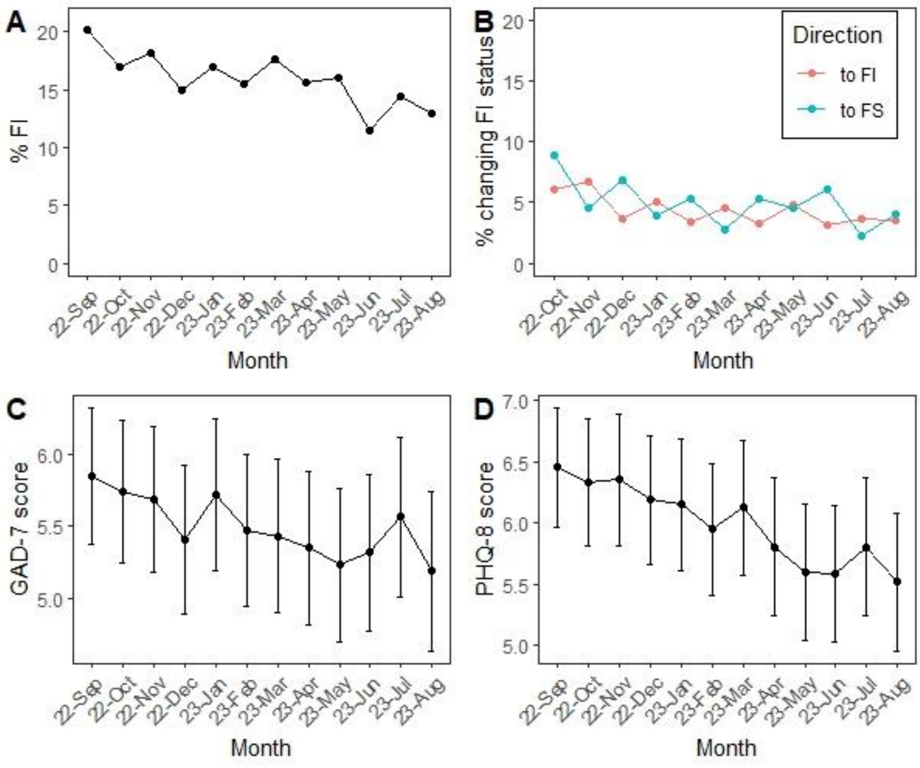


**Figure A.** Changes in food insecurity and mental health over the study. (A) Percentage of participants with food insecurity (i.e. an FI score > 0) by month. (B) Percentage of participants changing food-insecurity status by month. (C) Mean GAD-7 score by month. (D) Mean PHQ-8 score by month. Error bars represent 95% confidence intervals.
