# Supplementary material for: Does food insecurity cause anxiety and depression? Evidence from the changing cost of living study
Source: PLOS Ment Health. 2025 Jul 16;2(7):e0000320. doi: 10.1371/journal.pmen.0000320 (PMC12798610; doi:10.1371/journal.pmen.0000320)
Supplement: Attachment — Submitted filename: PMEN-D-25-00089 Reponse to reviewers.docx [file pmen.0000320.s003.docx]

**Response to reviewers PMEN-D-25-00089**

Journal requirements:

Please review your reference list to ensure that it is complete and correct.

**We confirm that I have checked the reference list and none of the cited articles has been retracted.**

**We have added the following references to list in course of revising our paper:**

**7. Ejiohuo O, Onyeaka H, Unegbu KC, Chikezie OG, Odeyemi OA, Lawal A, et al. Nourishing the Mind: How Food Security Influences Mental Wellbeing. Nutrients. 2024 Jan;16(4):501.**

**42. Ridley M, Rao G, Schilbach F, Patel V. Poverty, depression, and anxiety: Causal evidence and mechanisms. Science. 2020 Dec 11;370(6522):eaay0214.**

**We note that although a correction has been published to reference 42, the errors in the original do not change the conclusions.**

Reviewer #1

The analysis showed that women were more likely to experience worse mental health following food insecurity than men, and older people were less likely than young. This finding was not discussed further in the discussion section. Was it something to explore further, what could be the reason behind this?

**This comment arises from a misunderstanding on the part of the referee. We did not, as they suggest, explore the effect modification by either gender or age for the effects of food insecurity on mental health in the current study. To do this would have involved fitting interactions between food insecurity and gender or age in models 1-3. We did not do this because we had no hypotheses or predictions about the existence of such interactions. Models 1-3 included main effects of gender and age to control for well-established associations between these variables and mental health that could have biased our results. The effects of gender and age that we report in models 1-3 replicate effects found in many previous studies and, in our view do not warrant any additional discussion since they have no bearing on the aims of the current paper. We have added the following sentence to the results section (lines 279-281) to clarify the role of gender and age in models 1-3: “We additionally included fixed effects of gender and age to control for known associations between these variables and mental health.”**

Which also brings me to one methodology clarification- were all the participants unrelated in terms of sharing a household? Can it be studied whether in the same household, food insecurity was perceived differently by men and women or younger and older and mental health was affected differently? Please add comment to the discussion about this.

**The CCLS was not designed to study participants in the same household, so it is not possible to study these questions with the current dataset. While it is logically possible that some participants in CCLS could have come from the same household, we have no means of knowing if this was the case (and we assume that such instances are either rare of nonexistent). Participants were therefore treated as independent in our analyses. We have added this detail to the analysis section of the manuscript (lines 202-205). We do not feel that this point warrants any additional discussion.**

Line 107- better to say “poorer” mental health- “reduced” can be confused with reduced risk ..i.e. good mental health..

**We have changed “reduced” to “poorer” as suggested.**

Line 117 – as above, can you add info to clarify if these were all separate households, i.e the same household was not represented multiple times by different members of the household?

**See our response to the same question above.**

Table 5 - I suggest changing the wording in the title of Table 5 “within participants” -to “amongst participants”- to avoid confusion with within and between FI status

**To address this potential confusion we have changed the title of Table 5 to read: “Output of models predicting mean standardised anxiety and depression scores from food-insecurity status in the subset of participants who changed their status during the study.”**

Line 368 – subheading wording - up until here the analysis was focused on change in the scores on the 2 scales, here it is about clinical anxiety and depression- perhaps make it clearer in the subheading

**In line with this comment, we have changed the subheading to read: “The impact of eliminating food insecurity on clinically-relevant symptoms of anxiety and depression”.**

Line 395 - typo: “in” a panel dataset or “using” a panel dataset

**We’ve inserted “using” as suggested.**

Line 402 -statement “food-insecure months were associated with higher anxiety and depression..” – please clarify do you mean score or clinical

**We’ve clarified this sentence as follows: “Within the subset of participants whose food-insecurity status fluctuated during the study, food-insecure months were associated with more symptoms of both anxiety and depression than the participant’s personal averages for the study.”**

**Reviewer #2**

- The authors should note that anxiety and depression symptoms are different from anxiety and depression as a disorder. The authors should therefore briefly discuss this in the introduction and use the appropriate term throughout the manuscript (there is a lack of consistency in this) to ensure that it is clear to readers especially clinicians who might find the study's findings useful.

- The above should also be taken into consideration in the discussion and conclusion.

**We have gone through the paper and corrected this inconsistency throughout, changing “anxiety and depression” to “symptoms of anxiety and depression”. We have also changed “clinical cases” to “cases with clinically relevant symptom levels” to reflect the fact that GAD-7 and PHQ-8 scores of 10 or more do not say anything about whether a clinical diagnosis has been made.**

- From lines 162 - 163, could the authors provide a justification for focusing on within-individual variation and not between or both?

**We have provided the required justification for focusing on within-individual effects in paragraph 5 of the Introduction (lines 91-104). Isolating and testing the effects of fluctuations in food-insecurity status within individuals is also aim 3 of the study (lines 131-132). To remind readers of this we have reworded the sentence in the methods to which the referee refers to read: “Since food insecurity varied both between and within individuals and our stated aim was to focus on within-individual variation….”**

- In line 177 change to 'Anxiety symptoms...' as this is what the GAD questionnaire in this study accessed.

**Changed as suggested.**

- PHQ is used to access the severity of depressive disorders in individuals with depressive disorder. I imagine it is used to access depressive symptoms (which is one of its uses) in this study. This should therefore be reflected in line 181 that should be 'Depressive symptoms...'.

**Changed as suggested.**

- In line 540 does the term 'future mental health' refer to the one month prediction? The authors should clarify this statement especially because the following descriptions states that not more than one month was explored.

**We are referring to the one-month result here. To clarify this we have changed the relevant sentence to read: “In addition to showing that food-insecurity status predicted a participant’s concurrent mental health, we took advantage of the time series nature of the CCLS dataset to demonstrate that food-insecurity status also predicted future mental health in the following month.”**

- The authors should give examples of such effects in line 581.

**We have added citations to two example studies here (line 639).**

- I don't quite agree with the authors position in line 581 - 583. Anxiety and depression are responses due to varying and most often compounding factors including an individuals socioeconomic/environmental exposure, genetic and biological dysregulations. Treatment therefore are tailored based on individual needs and involves medications (drugs), psychotherapy, nutritional interventions, or a varying combination of these. Also, anxiety and depression can negatively impact physical and mental health and vice versa either directly or indirectly. These in most cases requires medications with drugs. Refer to the following studies for more insights: https://doi.org/10.3390/nu16040501,

https://doi.org/10.1183/09059180.00007813,

https://doi.org/10.1016/j.actpsy.2024.104285,

https://www.ncbi.nlm.nih.gov/books/NBK559078/#:~:text=Go%20to:,Etiology,low%20levels%20of%20serotonin%20metabolites.

**We thank the referee for this comment and the references. We agree that our conclusion was overstated and have modified it accordingly (lines 638 onwards). It now reads: “Together these findings strongly suggest that food insecurity causes acute negative effects on mental health. Our results therefore contribute to a growing literature documenting the social determinants of mental health (e.g., 30,42). Specifically, our analyses suggest that interventions to prevent food insecurity should produce a substantial and immediate reduction in the prevalence of clinically relevant symptoms of anxiety and depression in populations currently experiencing periodic food insecurity, supporting the case for non-pharmaceutical mental health treatments (43).**”

**We have incorporated a citation to Ejiohuo et al (2024) into our introduction since the review is highly relevant to the current paper.**

- The authors should discuss based on lines 579 - 580 if this prediction is possible beyond the one month time period. If yes, how, and if not, why.

**We have also added the following paragraph to the discussion section (lines 568-573): “In the current study, we did not explore temporal lags of more than one month due to the limitations of the dataset available. Therefore, our results say nothing about the longer-term effects on mental health (beyond one month) of experiencing a transition in food-insecurity status. Studies with both larger numbers of participants and longer time series of monthly within-individual measurements of food insecurity and mental health are required to address questions about longer-term effects of food insecurity.”**

- From lines 584 - 586, give examples of such interventions. You can refer to the following study for such interventions: <https://doi.org/10.3390/nu16040501>

**Thank you for this reference; we have added a citation to the paper (reference 7).**
